# Malaria risk factors in north-east Tanzania

**DOI:** 10.1186/1475-2875-10-98

**Published:** 2011-04-20

**Authors:** Peter Winskill, Mark Rowland, George Mtove, Robert C Malima, Matthew J Kirby

**Affiliations:** 1 London School of Hygiene and Tropical Medicine, Keppel Street, London, WC1E 7HT, UK; 2 Department of Infectious Disease Epidemiology, School of Public Health, Faculty of Medicine, Imperial College London, UK; 3 Pan-African Malaria Vector Research Consortium; 4 National Institute for Medical Resarch, Amani Centre, PO Box 81, Muheza, Tanga, Tanzania

## Abstract

**Background:**

Understanding the factors which determine a household's or individual's risk of malaria infection is important for targeting control interventions at all intensities of transmission. Malaria ecology in Tanzania appears to have reduced over recent years. This study investigated potential risk factors and clustering in face of changing infection dynamics.

**Methods:**

Household survey data were collected in villages of rural Muheza district. Children aged between six months and thirteen years were tested for presence of malaria parasites using microscopy. A multivariable logistic regression model was constructed to identify significant risk factors for children. Geographical information systems combined with global positioning data and spatial scan statistic analysis were used to identify clusters of malaria.

**Results:**

Using an insecticide-treated mosquito net of any type proved to be highly protective against malaria (OR 0.75, 95% CI 0.59-0.96). Children aged five to thirteen years were at higher risk of having malaria than those aged under five years (OR 1.71, 95% CI 1.01-2.91). The odds of malaria were less for females when compared to males (OR 0.62, 95% CI 0.39-0.98). Two spatial clusters of significantly increased malaria risk were identified in two out of five villages.

**Conclusions:**

This study provides evidence that recent declines in malaria transmission and prevalence may shift the age groups at risk of malaria infection to older children. Risk factor analysis provides support for universal coverage and targeting of long-lasting insecticide-treated nets (LLINs) to all age groups. Clustering of cases indicates heterogeneity of risk. Improved targeting of LLINs or additional supplementary control interventions to high risk clusters may improve outcomes and efficiency as malaria transmission continues to fall under intensified control.

## Background

Tanzania is heavily affected by malaria which is one of the leading causes of morbidity and mortality in the country [1], accounting for over 30% of the national disease burden [2]. In order to specifically tailor and improve prevention measures targeted against the disease it is important to obtain detailed knowledge of factors associated with increased risk of malaria. Identification of the specific risk factors in a locality may provide support for existing preventative measures or the introduction of new ones and can indicate areas in which prevention activities are currently under-utilized.

The identification and quantification of heterogeneity in disease prevalence across a geographical range provides scope for targeting prevention and treatment interventions at high-prevalence or high-risk areas [3,4]. This may in turn lead to increases in the equity, efficacy and cost effectiveness of interventions. Vector-borne diseases, such as malaria, are well suited to cluster analysis, which aims to delimit hotspots of high disease prevalence. The specific habits and limited range of the anopheline vectors of malaria aid efforts to resolve spatial clusters of the disease [5].

A number of studies have used cluster analysis to identify spatial and temporal hotspots of malaria transmission in other parts of Africa [6-9]. The epidemiology of the disease in eastern Africa appears to have changed in recent years, with marked declines in malaria transmission intensity, morbidity and mortality [10-14], making a study of this kind relevant and timely. As malaria declines, continued improvements of prevention and control interventions as well as treatment distribution may increasingly rely on accurate knowledge of risk factors and an ability to delimit high-risk areas.

This study aimed to investigate changes in malaria epidemiology in Muheza district by identifying significant household risk factors from individual, behavioural and house structural parameters. In addition, the study aimed to identify spatial clustering of malaria cases. It is intended that the study outcomes will inform targeting of interventions and treatment in the district and shed light on current epidemiological patterns.

## Methods

### Study area

The study was conducted within Muheza district in the Tanga region of North-East Tanzania (5°1'-5°8'S, 38°46'-38°56'E). A 2001 census recorded the district population as 1,636,280 [15]. Data were collected between June and August 2010, soon after the end of the traditional long rainy season. *Anopheles gambiae *s.l. has been shown to be the dominant vector in this region with abundance patterns strongly correlated with seasonal rainfall [16]. Malaria transmission usually peaks shortly after the end of the rainy season with prevalence in this region traditionally considered high. Previous studies have documented greater than 40% prevalence in children and young adults [17] and associated intensive, holoendemic transmission in the area [18,19].

All children participating in the study were aged between 6 months and 13 years and resided in twenty one rural hamlets within five villages: Mlingano, Mwungano, Kwalubuye, Kibaoni and Misozwe. Villages were selected based on three criteria: that they had a population of approximately 400 residents and/or 100 children, that they were not involved with any other research programme and that they were within a logistically feasible distance of the research centre in Muheza town. Three state-recognised drug dispensaries were located within the study area: one in Misozwe and two in Kibaoni. Over 90% of all household heads in the study worked as farmers, mainly at the subsistence level.

### Household survey and enumeration

Census data from 10,555 residents occupying 2,609 houses were collected during an enumeration round. Details were recorded of malaria prevention behaviours, socioeconomic status, house structure and location. GPS coordinates were taken from the front door of each residence using a GPS receiver (Garmin *e*trex legend^®^, Garmin International Inc. USA). Data were recorded using paper and personal digital assistants (PDAs) (HP iPAC 114 Classic, Hewlett-Packard Development Company, L.P. USA). Specific variables recorded were: individual-related: Age, gender, weight, height. Prevention behaviour-related: mosquito net use, type of mosquito net used (untreated, insecticide-treated net (ITN), LLIN), frequency of mosquito net use, number of holes in mosquito net, proximity of penned livestock and structurally-related: house elevation, house volume, wall and roof construction material, presence and size of eaves and the number of rooms, doors and windows.

Socioeconomic variables recorded included ownership of: a radio, a carved or iron bed, a cart, a bicycle, a car or motorbike and any livestock. A crude socioeconomic score (SES), ranging from 0 to 6, was calculated as the sum of positive responses to questions concerning the ownership of the aforementioned items. Body mass index (BMI) was calculated as weight/height^2^. The two age groups were based on observed differences in parasite prevalence of the two groups. All data were entered into a Microsoft Access database. The top and bottom 5% of values for each variable were extracted, double checked and verified.

### Malaria prevalence survey

Biometric measurements were taken from each child. Axillary temperature was taken using a digital thermometer and the parent/guardian was questioned about their child's recent history of fever. If axillary temperature was ≥37.5°C or a history of fever was reported then a rapid diagnostic test (RDT) was administered (Paracheck^® ^*Pf *device, Orchid Biomedical Systems, India) to test for *Plasmodium falciparum*-specific histidine rich protein II. Thick and thin blood films were made for all children. Slides were stained with Giemsa, and double read at Teule hospital, Muheza. Asexual stage parasites were counted per 200 white blood cells (WBC) and gametocytes were counted per 500 WBC. Anti-malarials (Coartem^®^, dispersable, artemether/lumefantrine 20 mg/120 mg) were given if RDT was positive.

### Statistical analysis

#### Risk factors

Statistical analysis and model building were performed using STATA software (version 11, College Station, TX, USA). All variables were analysed individually for an association with malaria risk using logistic regression. All variables showing evidence for a possible association with malaria risk (p < 0.15) were included in the preliminary main-effects multivariate logistic regression model. A stepwise backwards-elimination approach was then followed to exclude any variable that showed a lack of effect on malaria risk (p > 0.05). Models were multilevel to adjust for possible clustering of cases within village and households; this gave reduced weighting to each subsequent malaria positive child recorded from a village or household after the first. For multivariable analysis untreated ITN and no mosquito net use were combined, as was insecticide-treated ITN and LLIN use. Wald tests were used to analyse the effect of removing each non-significant variable from the model. Possible interaction terms were also considered before a variable was dropped.

#### Clusters

Spatial analysis was performed to look for possible clustering of cases across households. A Kuldorff spatial scan statistic was obtained using the Bernoulli model [20,21] and SaTScan software (SaTScan, version 8.2.1). The software applies an infinite number of circular windows, which are plastic in both position and size, across the study area. Each distinct circle represents a possible cluster. A likelihood ratio test compares the observed prevalence of disease within the circle to the expected prevalence across the entire range to identify significant clusters of disease, providing relative risk and *p-*values for any clusters identified. The model was run with a maximum cluster size of 50% of the total population and *p*-values generated across 999 Monte Carlo replications.

#### Geographical

House locations were displayed using the GPS data and a geographical information system (GIS) (ArcMap, version 9.2, CA, USA). Data on administrative boundaries [22] were downloaded and added to the map as a layer. Clustering analysis statistics were also displayed and inspected using the ArcMap GIS.

### Ethical approval

Ethical approval for this study was granted from the London School of Hygiene and Tropical Medicine (LSHTM) ethics committee and the National Institute for Medical Research (NIMR) Medical Research Coordination Committee (NIMR/HQ/R.8a/Vol.IX/928). Each parent/guardian and their children were informed of the purpose of the study and the nature of the clinical prevalence work. Verbal and written consent was obtained from all study participants.

## Results

A total of 1438 children were included in the study, 728 (51%) male, 702 (49%) female and 8 missing values. Overall *P. falciparum *prevalence was 14.5% (95% CI 12.7-16.3). 43.6% (95% CI 38.5-48.8) of children did not have a mosquito net, 50.6% (95% CI 45.4- 55.7) had an LLIN, 1.1% (95% CI 0.5-2.5) an insecticide-treated net and 4.7% (95% CI 3.1-7.0) an untreated net. Children in the younger age group (6 months-4 years) were more likely to sleep under an insecticide-treated mosquito net than children in the older age group (5-13 years) (χ^2 ^= 77.5, *p *< 0.001). There was no difference in the utilisation of mosquito nets between male and female children (χ^2 ^= 0.09, *p *= 0.8).

### Univariate analysis

Results of univariate analysis, after adjusting for possible village- and house-level clustering of cases, are shown in Table 1. Both age and gender was significantly associated with malaria risk. Children in the older age group (5-13 years) and males were associated with a significant increase in the odds of malaria infection. Use of an insecticide-treated mosquito net was associated with a decrease in the odds of malaria infection. As there was some evidence of an association between open eaves and malaria risk and also SES and malaria risk, these variables were also used in the multivariate modelling.

**Table 1:** Univariate analysis of variables examined as potential risk factors for malaria in children

Variable	n	Cases (%)	OR (95% CI)*	*p*-value
**Individual**				
Age group				
6 months-4 years	518	48 (9.3)	1.0	-
5-13 years	919	160 (17.4)	2.06 (1.26-3.38)	0.015
Sex				
Male	727	123 (16.9)	1.0	-
Female	702	85 (12.1)	0.68 (0.44-1.05)	0.068
Body mass index (BMI) (per additional unit)	1436	208 (14.5)	0.97 (0.90-1.05)	0.40
Socioeconomic status (per additional point score)	1437	208 (14.5)	0.87 (0.72-1.06)	0.12
Number of co-residents (per additional resident)	1432	207 (14.5)	1.05 (0.94-1.17)	0.26
**Prevention behaviour**				
Mosquito Net use				
None	556	109 (19.6)	1.0	-
Untreated mosquito net	59	8 (13.6)	0.64 (0.22-1.88)	0.32
Insecticide-treated mosquito net	659	70 (10.6)	0.49 (0.30-0.78)	0.013
Frequency of net use				
Every night - seasonal	235	29 (12.3)	1.0	-
Every night - year round	625	66 (10.6)	0.84 (0.42-1.68)	0.52
Number of holes in net (per additional hole)	875	98 (11.2)	0.98 (0.91-1.06)	0.51
Proximity of penned livestock				
No livestock penned < 20 m from house	835	131 (15.7)	1.0	-
Livestock penned < 20 m from house	594	77 (13.0)	0.80 (0.50-1.27)	0.25
**House related**				
Elevation	1393	204 (14.6)	1.00 (1.00-1.01)	0.28
Wall material				
Cement	327	41 (12.5)	1.0	-
Mud	1089	161 (14.8)	1.21 (0.68-2.14)	0.41
Wood	9	3 (33.3)	3.49 (0.31-39.29)	0.23
Other	5	1 (20)	1.74 (0.12-24.55)	0.59
Roof material				
Metal sheet	722	100 (13.9)	1.0	-
Grass	75	6 (8.0)	0.54 (0.14-2.06)	0.27
Coconut palm	588	97 (16.5)	1.23 (0.77-1.97)	0.29
Other	42	1 (2.4)	0.15 (0.01-2.52)	0.14
Eaves				
Closed	164	16 (9.8)	1.0	-
Open	1261	189 (15.0)	1.63 (0.73-3.63)	0.17
House size (m^2^)	1428	207 (14.5)	0.99 (0.99-1.00)	0.65
Number of rooms (per additional room)	1429	208 (14.6)	0.87 (0.68-1.11)	0.19
Number of windows (per additional window)	1426	207 (14.5)	0.86 (0.75-0.99)	0.038
Number of doors (per additional door)	1430	208 (14.5)	0.98 (0.66-1.46)	0.90

* Robust confidence intervals adjusted for within village and within household clustering of cases

### Multivariate analysis

Multivariate logistic regression analysis with an adjustment for within village and household clustering indicated that there were three variables significantly associated with malaria risk (Table 2).

**Table 2:** Logistic regression model of significant variables associated with malaria risk in children

Variable	Adjusted OR (95% CI)*	*p*-value
**Insecticide-treated mosquito net use**		
No	1.0	-
Yes	0.75 (0.59-0.96)	0.030
**Age group**		
6 months-4 years	1.0	-
5-13 years	1.71 (1.01-2.91)	0.048
**Sex**		
Male	1.0	-
Female	0.62 (0.39-0.98)	0.044

* Robust confidence intervals adjusted for within village and within household clustering of cases

Risk of infection was significantly higher in children of five to thirteen years of age, compared to those aged six months to four years. There was strong evidence that sleeping under an insecticide-treated mosquito net was highly protective. The risk of malaria was lower for female than for males. There was no association between malaria risk and wall or roof construction material. There was no evidence of interaction between variables.

### Cluster analysis

**Figure 1:**
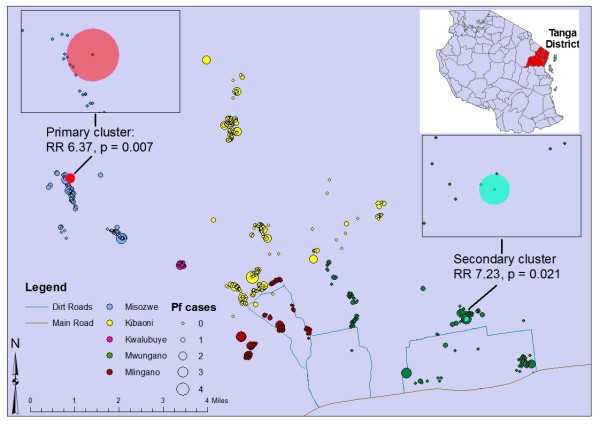
**Spatial clustering of malaria cases**.

Spatial scan statistic indicated two distinct spatial clusters of malaria cases (Figure 1). In these areas the observed number of cases was significantly higher than expected (Table 3). The primary cluster consisting of four houses was located in Misozwe. A secondary, smaller cluster of two houses was identified in Mwungano.

**Table 3:** Details of two distinct significant spatial clusters of malaria in children

Cluster	Radius (Km)	Population (n)	Observed cases	Expected cases	Relative risk (RR)	*p*-value
**Primary**	0.16	8	7	1.1	6.4	0.007
**Secondary**	0.04	5	5	0.7	7.2	0.021

## Discussion

Risk factor analysis performed for this study identified a number of variables that, alone or in unison, would affect a child's risk of malaria. The child's age, gender and insecticide-treated mosquito net use were each associated with malaria risk. Use of insecticide-treated mosquito nets was highly protective against malaria whereas untreated nets were not. Older children, aged 5-13 years, and males had increased odds of malaria. Cluster analysis attempted to identify spatial hotspots of malaria where a child's risk of malaria was higher than expected for the study population as a whole. Two significant clusters of malaria were identified in two separate villages.

Age remained an important factor within the model, with older age groups being exposed to a higher risk of malaria. This finding is of particular interest as historically in Tanzania prevalence has peaked in younger age groups [23-25]. Part of this pattern is explained by the relationship between age and insecticide-treated mosquito net use. Children in the younger age group were significantly more likely to sleep under insecticide-treated mosquito nets which, in this study and many others across Africa [26], have proven to be highly protective against malaria. The observed age-prevalence patterns may be partially driven by overall increases in LLIN coverage in Tanzania [27]; shifts in the age of peak prevalence towards older children have been observed with increases in mosquito net coverage [28]. Similar shifts may be observed with reductions in the entomological inoculation rate (EIR) and the force of infection [29,30] leading children to be exposed to infective malaria inoculations less frequently meaning infections are acquired later in life. However, age-specific parasite-prevalence patterns have been demonstrated across a range of transmission intensities in this region of Tanzania before [17], suggesting that a drop in transmission intensity would be associated with reduced malaria-associated morbidity in all age groups and not a pronounced shift in age prevalence peaks. In this case a continued commitment to LLIN distribution would see further benefits for local communities [31] without risking potential changes of age-associated patterns in the clinical outcomes of malaria [32,33]. Preferential provision of mosquito net coverage to the younger age group will remain the most beneficial distribution of scarce resources as the burden of malaria-associated deaths and morbidity is likely to remain highest in the very young [34].

In this study male children were at higher risk of malaria, possibly indicating that males were associated with high exposure behaviour, although insecticide-treated mosquito net coverage was not gender-skewed. Gender as a risk factor is likely to be linked to exposure, inherent and cultural factors and has inconsistent associations with malaria risk [35,36]. Due to the nocturnal habit of the anopheline vector, data concerning the time at which children went to bed would have been an interesting variable to examine in relation to the observed age- and gender-related risk patterns. A previous study in Muheza district showed a contrasting, though non-significant, reduced risk of malaria in male children [37]. Other studies in Africa have shown no differences between gender and malaria risk [8,38] and risk fluctuating between sexes across a number of seasons [39] suggesting that distribution of risk is both spatially and temporally heterogeneous.

The protective effect of insecticide-treated mosquito net use shown in this study adds to the vast body of evidence supporting the efficacy and effectiveness of insecticide-treated mosquito nets for protection against malaria and other vector-borne diseases in this setting [26]. In this population approximately one half of the children in the study did not own or use an insecticide-treated mosquito net, increasing their risk of malaria by around 25%. This far from universal coverage is especially poignant when Tanzania is considered to be one of the continent's success stories for mass ITN and LLIN distribution [40,41]. It also gives weight to the importance in understanding the full range of risk factors for malaria in this region. This result provides scope for further reductions in transmission with scaling up of LLIN distribution and the goal of universal coverage [42].

Increased ability to target interventions in the Tanga region, a resource-limited setting, is vital. Identifying high-risk areas for disease allows policy to be tailored to give highest priority for LLIN distribution to those most at threat. An aggregation of cases within clusters may be somewhat driven by an elevated number of higher risk demographic groups residing within houses located in the clusters. Other factors not examined during this study but known to be important could also be rendering these areas as high risk [6,8,38,43,44]. Specifically, the addition of entomological data regarding the vicinity of villages and clusters to anopheline breeding sites would have provided further insight into the biological mechanisms associated with infection risk. Unfortunately there was insufficient budget to complete an enotomological risk factor analysis but it will be important to complete one for verification in future studies. Further information concerning residents' access to the health facilities available would also be useful to allow targeted improvements to treatment availability in the area. Identification of remaining high-risk areas may become more important if current declines in transmission continue, allowing resources to be targeted to areas that remain at high risk or where declines in transmission are less pronounced.

## Conclusions

Identifying potential high-risk areas and the mechanisms by which the risk arises can have implications for the whole range of malaria-focussed activities, from increased surveillance to targeted interventions and treatment. In a low-income setting, such as rural Tanzania, any advances in the cost-efficiency and equitability of disease control are crucial.

Malaria epidemiology in the Tanga region has not been static over recent years [10-14]. This study provides evidence that recent declines in malaria transmission and prevalence may be affecting the age profile of malaria prevalence among children in the Muheza district, shifting the peak in risk of malaria infection to older age groups.

Clustering analysis, when combined with knowledge of specific risk factors, will assist targeting of intervention measures to specific high-risk zones which could reduce costs, increase efficacy and improve the equity of control measures for the population in question. The ability to define high-risk areas for targeting of interventions in a newly emerging region of reduced transmission intensity is vital for local elimination of transmission and the rational distribution of control interventions.

## Competing interests

The authors declare that they have no competing interests.

## Authors' contributions

The study was designed by MK, PW and MR. PW, MK, GM and RM collected the data. PW, MK and MR drafted the manuscript. All authors read and approved the final manuscript.
